# Molecular Factors Mediating Neural Cell Plasticity Changes in Dementia Brain Diseases

**DOI:** 10.1155/2021/8834645

**Published:** 2021-03-29

**Authors:** Wojciech Kozubski, Kevin Ong, Wioletta Waleszczyk, Matthew Zabel, Jolanta Dorszewska

**Affiliations:** ^1^Chair and Department of Neurology, Poznan University of Medical Sciences, Poland; ^2^General Medicine Department, Armadale Health Service, Mount Nasura, Australia; ^3^Laboratory of Visual Neurobiology, Department of Neurophysiology, Nencki Institute of Experimental Biology, PAS, Warsaw, Poland; ^4^College of Medicine, California Northstate University, CA, USA; ^5^Laboratory of Neurobiology, Department of Neurology, Poznan University of Medical Sciences, Poznan, Poland

## Abstract

Neural plasticity—the ability to alter a neuronal response to environmental stimuli—is an important factor in learning and memory. Short-term synaptic plasticity and long-term synaptic plasticity, including long-term potentiation and long-term depression, are the most-characterized models of learning and memory at the molecular and cellular level. These processes are often disrupted by neurodegeneration-induced dementias. Alzheimer's disease (AD) accounts for 50% of cases of dementia. Vascular dementia (VaD), Parkinson's disease dementia (PDD), dementia with Lewy bodies (DLB), and frontotemporal dementia (FTD) constitute much of the remaining cases. While vascular lesions are the principal cause of VaD, neurodegenerative processes have been established as etiological agents of many dementia diseases. Chief among such processes is the deposition of pathological protein aggregates *in vivo* including *β*-amyloid deposition in AD, the formation of neurofibrillary tangles in AD and FTD, and the accumulation of Lewy bodies composed of *α*-synuclein aggregates in DLB and PDD. The main symptoms of dementia are cognitive decline and memory and learning impairment. Nonetheless, accurate diagnoses of neurodegenerative diseases can be difficult due to overlapping clinical symptoms and the diverse locations of cortical lesions. Still, new neuroimaging and molecular biomarkers have improved clinicians' diagnostic capabilities in the context of dementia and may lead to the development of more effective treatments. Both genetic and environmental factors may lead to the aggregation of pathological proteins and altered levels of cytokines, such that can trigger the formation of proinflammatory immunological phenotypes. This cascade of pathological changes provides fertile ground for the development of neural plasticity disorders and dementias. Available pharmacotherapy and disease-modifying therapies currently in clinical trials may modulate synaptic plasticity to mitigate the effects neuropathological changes have on cognitive function, memory, and learning. In this article, we review the neural plasticity changes seen in common neurodegenerative diseases from pathophysiological and clinical points of view and highlight potential molecular targets of disease-modifying therapies.

## 1. Introduction

The impairment of cognitive functions, memory deficits, and the deterioration of learning processes that appear in neurodegenerative diseases have been linked to neural plasticity dysfunction in the central nervous system (CNS) [1]. Neural plasticity is defined as the ability to alter a neuronal response to environmental stimuli and is the foundation of learning and memory. Cognitive processes that create lasting memories are based on the mechanism of long-term synaptic potentiation that reflects the inherent plasticity of the brain [1–5].

Neuronal plasticity is a basic property of the nervous system, which ensures both the brain's normal functioning and its capacity for partial regeneration. Plasticity encompasses both synaptogenesis during early development and compensatory and corrective plasticity in the adult brain. Short-term synaptic plasticity and long-lasting synaptic plasticity, including long-term potentiation (LTP), long-term depression (LTD), and homeostatic plasticity, are the best-developed models of learning and memory at the molecular and cellular levels [6–8].

Short-term plasticity is thought to occur by three likely interrelated processes: facilitation, potentiation, and augmentation [2]. While the latter two of these remain poorly characterized, facilitation involves increased synaptic vesicle exocytosis secondary to an accumulation of Ca^2+^ ions at the presynaptic following several action potentials that occur in rapid succession (several ms). Increased transient levels of Ca^2+^ are attributed primarily to the fact that Ca^2+^ influx through presynaptic voltage-gated Ca^2+^ channels occurs faster than Ca^2+^ efflux via active processes but also to the saturation of Ca^2+^ buffers (e.g., calbindin) following the first action potential, which leads to increased free Ca^2+^ upon subsequent axonal depolarization events. With increased synaptic vesicle exocytosis at excitatory synapses come larger excitatory postsynaptic potentials (EPSPs). Intelligibly, however, depletion of the synaptic vesicle pool may occur with high-frequency stimulation. This process has been named synaptic depression and directly opposes facilitation [6, 7].

While the mechanisms underlying short-term plasticity transpire largely at the presynapse, the signal transduction pathways associated with long-term plasticity occur postsynaptically [6–8]. The first major advance in understanding the molecular underpinnings of LTP came in the mid-1980s when it was found that antagonists of the N-methyl D-aspartate (NMDA) subtype of ionotropic glutamate receptors inhibited the process at hippocampal glutamatergic synapses. Thenceforth, several elegant studies elucidated that the NMDA receptors (NMDARs) responsible for the Ca^2+^ influx at the postsynapse remain blocked by a magnesium ion at more polarized membrane potentials. This magnesium blockade is reversed once a train of presynaptic action potentials generates sufficient glutamate in the synaptic cleft to strongly depolarize the postsynaptic membrane via *α*-amino-3-hydroxy-5-methyl-4-isoxazole propionic acid (AMPA) receptor- (AMPAR-) mediated sodium influx. Postsynaptically, Ca^2+^ activates calmodulin (CaM) kinase II (CaMKII) and protein kinase C (PKC), which increase membrane expression of AMPARs, effectively *potentiating* the process. In late-stage LTP (on the order of hours), Ca^2+^-driven regulation of adenylyl cyclase may activate PKA, which in turn drives cAMP-response element binding protein- (CREB-) transactivation of genes involved in synaptic remodeling (e.g., dendritic spine formation). Interestingly, low-frequency firing of action potentials in the presynaptic neuron does not merely lessen the probability of LTP but results in LTD at hippocampal glutamatergic synapses [2]. Indeed, seminal studies have demonstrated that a slow increase in postsynaptic levels of Ca^2+^ activates phosphatases instead of kinases, leading to AMPAR internalization.

In addition to glutamate, synaptic plasticity may be regulated by acetylcholine (ACh), dopamine (DA), gamma-aminobutyric acid (GABA), glycine, norepinephrine (NE), serotonin (5-HT), and other neurotransmitters [3, 9]. In the aging brain, the levels of these neuromodulators undergo significant changes [4]. Increased glutamate levels have been attributed to defects in proteins responsible for uptake of the neurotransmitter including the glutamate transporter GLT1 and the astrocyte-expressed excitatory amino acid transporter EAAT2, and those regulating glutamate turnover, such as glutamine synthase. Such defects may be caused by reactive oxygen species (ROS). Excessive glutamate leads to increased ROS-generating enzyme activity via enhanced stimulation of NMDA receptors and Ca^2+^ influx into neurons [1, 10]. *In vivo*, ROS are formed as a result of intracellular metabolic processes involving oxygen. The most reactive form of oxygen is the hydroxyl radical, which damages macromolecular compounds such as lipids, proteins, and nucleic acids. Oxidative damage sustained by DNA and not repaired generates mutagens, which exacerbate the effects of excitotoxicity and promote neurodegeneration [1].

The dysregulated release of certain cytokines including interleukin-1*β* (IL-1*β*) is also implicated in aberrant glutamatergic signaling [11]. IL-1*β* inhibits glutamate release by acting upon the transcription factors c-Jun N-terminal kinase (JNK), p38, and extracellular signal-regulated kinase (ERK) [12–14]. Interestingly, it has been shown that supplementation with ROS scavengers leads to the improvement of cognitive function by normalizing the levels of ROS, IL-1*β*, JNK, p38, and activity of superoxide dismutase (SOD) [15–17].

Mild cognitive impairment (MCI) in normal aging is primarily deficits in working memory. Observed intellectual deterioration symptoms include difficulties with carrying out complex cognitive tasks such as understanding of language, reading, learning, or reasoning [18]. In the aging brain, change of memory and cognitive functions is the result of the disturbances of Ca^2+^ homeostasis and metabolic dysfunction of old neurons and mitochondria. It may also result from incorrect expression of growth factors, such as glial-cell-derived neurotrophic factor (GDNF), nerve growth factor (NGF), and brain-derived neurotrophic factor (BDNF) [19]. Changes in the levels of these factors and synaptic proteins involved in the structural plasticity of axons and dendrites may alter surface contact between neurons [20]. Disturbed contact between neurons interfere with neuronal networks, leading to senile changes and the development of dementia [21].

## 2. Clinical Symptoms of Common Neurodegenerative Diseases

Common causes of dementia include Alzheimer's disease (AD) and vascular dementia (VaD), Parkinson's disease (PD) dementia (PDD), dementia with Lewy bodies (DLB), and frontotemporal dementia (FTD). Neurodegenerative diseases which cause dementia often comprise the clinical picture of the impairment of cognitive efficiency and personality and emotional changes including psychopathological disorders. The clinical picture may also include other neurological symptoms like parkinsonism [22]. These clinical symptoms are often the result of neural plasticity disorders.

AD is the most common cause of dementia in advanced age, accounting for 50% of all dementia cases [23]. The neuropathological hallmarks of AD are deposits of extracellular *β*-amyloid (A*β*) plaques and intracellular tau aggregates. While AD has an inescapable, progressive course, the clinical presentation varies at different stages of the disease. Symptoms at early stages may consist of episodic memory decline, visuospatial disorientation, decline of executive functions, and negative affect [24]. About 70% of patients with AD neuropathology experience intellectual decline, but the decline is often too mild to clinically qualify as dementia [25]. As the disease progress, AD patients present with disruption of other types of memory (working and semantic memories); language disturbances like verbal fluency and word finding difficulties; a decline of praxis (the ability to make purposeful movements), apraxia (inability or difficulty to carry out a command), and dyspraxia (a coordination impairment); and difficulties with visuospatial skills [26]. Patients with advanced AD may present with a whole variety of behavioral or neuropsychiatric symptoms including apathy; depression; euphoria; perception alterations such as hallucinations, delusions, and misidentifications (Capgras syndrome); and inversion of circadian rhythm (with night agitation) [27, 28]. In frontal variant AD, the behavioral changes are similar to those encountered in FTD (discussed later). In the late stage of the disease, patients are mute and bedridden. The disease shortens life expectancy, and the most common causes of death are pneumonia and urosepsis [26].

Both PD and PDD are neurodegenerative disorders with Lewy bodies (LB) which consist mainly of *α*-synuclein (ASN) [29]. DLB, wherein LB are present in both cortical and subcortical brain regions, is considered as a distinct entity but nonetheless has some clinical and pathological overlap with both AD and PD. The disease is believed to be relatively rare, constituting 4-16% of all dementias; however, it seems to be underdiagnosed [30]. Clinical presentation resembles, to some extent, the dementia of the Alzheimer's type; however, significant differences may be observed including fluctuating cognitive status and compromised attention span. Importantly, psychotic symptoms are present in up to 75% of DLB patients. The psychiatric symptoms are mostly those of delusion and involve mis- and auto-mis-identifications, paranoid beliefs, and vivid, colourful hallucinations; however, patients may remain partly self-aware [31]. Patients with DLB often display rapid eye movement (REM) sleep behavior disorder (RBD) with dream enactment and loss of skeletal muscle tone that result in sleep fragmentation and decreased sleep quality [32]. More than 70% of patients with DLB present with motor signs of parkinsonism, with symmetrical rigidity, bradykinesia, and tremor, which, unlike that seen in PD, is largely positional and intentional. The parkinsonian symptoms are poorly responsive to L-dopa treatment [33] and typically appear within one year from the time intellectual decline is first noticed. Most patients with DLB show hypersensitivity to neuroleptic/antipsychotic drugs, especially to phenothiazine and butyrophenone derivatives, with rapid development of parkinsonian symptoms and signs when administered [34].

PDD, where LB are present in the subcortical basal ganglia, is characterized by a progressive motor decline of the parkinsonian type, diminishing cognitive performance, episodic memory deficits, and loss of executive, attentional, and visuospatial skills. Dementia in PD is assessed using clinical criteria according to the Diagnostic and Statistical Manual of Mental Disorders (DSM) [35, 36]. This symptom occurs in 20-40% of PD patients [37, 38]. In addition to the aforementioned symptoms, patients may present with behavioral disturbances that may be provoked by visual hallucinations and agitation during the REM phase of sleep (RBD). PD MCI is the most important risk factor for PDD, together with advanced age, increasing motor deficits, postural instability, and early development of psychiatric symptoms (mostly hallucinations) [39].

FTD is characterized by a loss of neurons in the more superficial cortical layers of mainly the frontal and temporal lobes. Primary frontotemporal neurodegenerative dementia may be subdivided into three main clinical categories: frontal variant FTD, primary progressive aphasia (PPA), and semantic dementia (SD) with frontal and temporal lobe involvement [23]. FTD generally affects patients in the age range of 45-65 years, while semantic dementia usually starts later, around the age of 70 years. 30 to 50% of FTD cases are familial [40]. Personality changes and behavioral disinhibition dominate in frontal variants. The beginning of symptoms is insidious: patients usually present with symptoms and signs of frontal lobe involvement, namely, overactivity and restlessness [41]. As the disease progresses, apathy with lack of motivation and initiative ensues and patients may exhibit socially unacceptable behavior, self-centredness, emotional lability, and impulsivity. Obsessive, repetitive, and compulsive behaviors are relatively common in all stages of the disease [42]. Early symptoms of PPA include incoherent language and nonfluent aphasia, with noticeable speech hesitation that contains numerous semantic and phonemic errors [23]. Word finding difficulties and repetition of sounds, words, and phrases are readily observable. Social skills are persevered until other frontal symptoms emerge [43]. On rare occasions, the nonfluent form of PPA may present with early impairment in comprehension but with preservation of word articulation [23]. SD is characterized by loss of semantic knowledge; that is, the patient loses conception of language and word meaning. Here, speech is fluent, articulate, and grammatically correct but full of paraphasia and substantively lacking. Patients with SD might show disinhibited behavior resembling frontal lobe variant FTD [44].

VaD arises from ischemic injuries such as hemorrhage and hypoperfusion. AD and VaD often coexist and have overlapping neuropathological features. These include the presence of A*β* plaques, neurofibrillary pathology, and cholinergic deficits but to a lesser extent in VaD than in AD. Clinical symptoms due to VaD can include confusion, language deficits, anxiety and agitation, gait disturbances, and progressive cognitive impairment, memory loss, and thinking or speech problems. All these symptoms are also found in patients with AD [45, 46].

## 3. Central Changes Associated with Clinical Symptoms of Dementia

The hippocampus is a bilateral structure located in the medial temporal lobe of the brain, which, together with the nearby dentate gyrus, entorhinal cortex, and rootstock forms the so-called hippocampal formation. This compound cortical structure is responsible for spatial orientation, memory consolidation, integration, recollection, and learning. Damage of the hippocampal formation, which manifests mainly with impairment of memory processes, is characteristic of neurodegenerative diseases like AD. Neuronal loss occurs in the rootstock and the dentate gyrus during physiological aging, while in AD atrophy is also noticeable in CA1 interneurons [47, 48]. The consequences of the said pathological changes accord with the observations of Kramer et al. [49], who noted an association between reduction in hippocampal gray matter volume and episodic memory impairment, where episodic memory refers to the spatiotemporal codification of experiences and the ability to recall them [50]. With age, additional changes occur in the brain contributing to the reduction of hippocampal gray matter volume including a decreased number of dendritic spines, cellular hypoplasia, and poorer myelination of fibers leading to slower transmission of action potential [51, 52].

Anterograde amnesia may characterize early stage AD; however, long-term memories of past events, which are thought to be stored in the anterior cortex and other cortical areas, remain intact [53]. This may be attributed to the hippocampal dysfunction that occurs at the beginning of AD [52–55]. Later in the course of AD, the anterior regions of the cortex are at risk of deterioration [52, 54].

Spatiotemporal memory encoding involves communication between the entorhinal cortex and association cortices. Despite functional interdigitation, both intra- and extrahippocampal circuits associated with this pathway have been identified. The former, termed the perforant pathway, represents the major input pathway to the hippocampus, while the latter uses the cingulum, a tract forming the core of the cingulate gyrus, as a conduit. The perforant pathway consists of axons arising predominantly from the second and third layers of the entorhinal cortex that synapse onto the granule cells of the dentate gyrus, and, to a lesser extent, CA1 and CA3 pyramidal cells, and nerve cells of the rootstock. Damage here, which occurs typically in AD, may isolate the hippocampus from cortically expedited processes in memory integration, leading to memory impairment. It should be noted that the order of the occurrence of neurofibrillary changes in the neocortex is not random. Usually, changes in associative cortices occur earlier than in the motor and somatosensory projection cortices, leading first to memory impairment, then reduction of intellectual abilities, and finally to impaired perception of stimuli [56, 57].

Interestingly, the hippocampus has also been implicated in the dopaminergic system. Although changes in the old brain to DA metabolism are subject to large individual variation [58], aging has been associated with decreased expression of the D2 receptor in the frontal cortex, hippocampus, amygdala, and thalamus [59]. Other reports have documented symptoms reflective of increased dopaminergic transmission in adult experimental animals bearing damaged hippocampi [60]. Specifically, it seems that the ventral hippocampus is involved in the control of dopaminergic system tone; however, differences in motor activity between control and experimental animals and the amount of DA released were not correlated [61]. Such results may indicate that the symptoms of increased dopaminergic transmission in animals with damaged hippocampi are not associated with an increase in the release of DA from presynaptic endings but rather with other mechanisms [62].

## 4. How and What Neuroimaging Can Inform Us about Neural Plasticity in the Aging Brain

Currently, a definitive diagnosis of AD is established via *post mortem* examination [63]. Jack et al. [64] proposed a new definition of AD in living people based upon the detection of certain biomarkers *in vivo*. AD biomarkers include A*β* peptide deposition, cerebral atrophy, hypometabolism, and tau aggregation forming neurofibrillary tangles (NFTs) (Table 1). It has been shown that the presence of these AD biomarkers serves both as a benchmark and indictor of the state of neurodegeneration encompassing neuronal damage, a reduction in the number of neocortical synapses, and cognitive loss [65, 66]. Synaptic loss, which can have a multifactorial etiology, closely correlates with impaired cognitive performance [64, 67].

**Figure 1:**
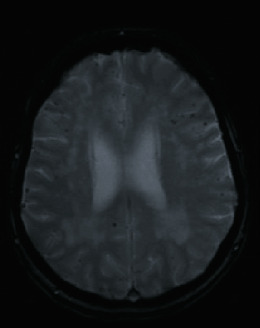
MRI (Gradient echo). 67F, presented with cognitive deficits which were first noticed by close relatives six years prior and initially thought to be due to severe depression. MMSE 18/30, ACER 58/100. MRI demonstrated extensive cerebral amyloid angiopathy.

**Figure 2:**
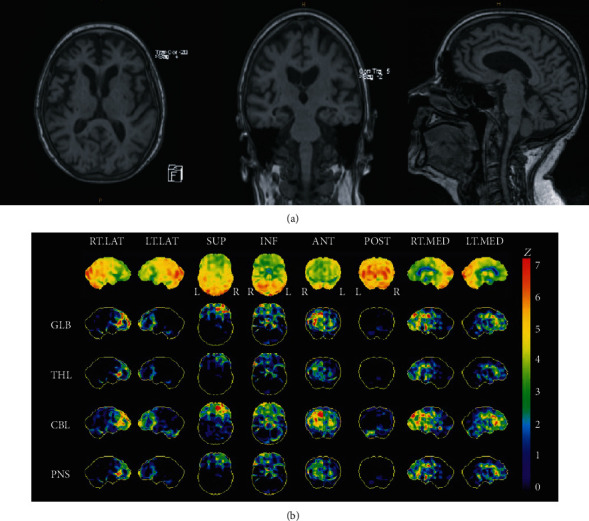
84F, independent with activities of daily living, MMSE 30/30, ACER 84/100, but occasional odd behaviors noted by children in the recent weeks. Diagnosed with early frontotemporal dementia. (a) Brain MRI—moderate generalized enlargement of the ventricles and surface CSF spaces, particularly affecting the frontal lobes bilaterally. Mild presumed microangiopathic changes and tiny old scattered lacunes. No features of remote microhemorrhages. (b) Brain SPECT—decreased activity is seen predominantly at both frontal lobes. There is also involvement of the anterior and mesial temporal lobes.

Current neuroimaging techniques encompass both structural imaging with brain computed tomography (CT) or magnetic resonance imaging (MRI) (Figures 1 and 2(a)) and molecular imaging with brain single-photon emission computed tomography (SPECT) (Figure 2(b)) or positron emission tomography (PET). Neuroimaging is relatively expensive, and access to the associated equipment varies, even in developed countries. These factors and other practical considerations may influence which neuroimaging modality is chosen in neuroimaging studies. That all conventional neuroimaging approaches have inherent limitations is concomitant to the fact that a particular technique will confer particular diagnostic advantages. For example, A*β* PET imaging is specific for detecting cerebral A*β* burden *in vivo*, whereas CT is specific in examining for brain atrophy and strokes. Finally, due to the heterogeneity of the ageing population, the cognitive impact of cerebral buffering mechanisms is generally subtle and nebulous.

Conventional dementia neuroimaging biomarker studies are generally observational, hypothesis driven, or for prove-of-concept. Neuroimaging studies have shown that people with neurodegenerative changes feature diminished cognitive anatomico-functional reserves, an intelligible conclusion given that clinical symptoms in illness like PD do not manifest until 50–70% of nigral dopaminergic neurons have been lost. Cabeza et al. [68] noted that PET and functional MRI were able to detect specific differences in patterns of activity between young and older adults performing a cognitive task, such as a word recall test. Specifically, the activity of the prefrontal cortex (PFC) in individuals performing the cognitive task was more unilateral in both young adults and poorly performing older adults, while the activity of the PFC in older adults who performed superiorly showed a more bilateral pattern. The obtained results suggest that in older adults, maintaining cognitive function may require neuronal circuit remodeling, such that is contingent on neural plasticity; however, this hypothesis requires further research.

Poor neuroimaging technique selection is intimately connected with decreased diagnostic precision and accuracy. Leveraging conventional neuroimaging techniques with novel approaches might enable us to detect mechanisms that buffer certain neurodegenerative changes and prevent symptoms from manifesting at early disease stages. For example, by applying Cox regression analyses, we demonstrated that increasing A*β* burden as detected by serial florbetaben PET scans in MCI volunteers predicted progression to dementia, irrespective of disease context [69]. Indeed, A*β* oligomers may be responsible for the deterioration of the cognitive reserve, while the dense A*β* plaques formed from them may constitute a kind of protective mechanism [70].

Appreciation for neural plasticity at both molecular and intercellular levels opens new corridors that might lead toward clinically meaningful neurodegenerative disease therapies.

## 5. Molecular Basis of Neurodegenerative Diseases and Their Impact on Synaptic Plasticity

### 5.1. Pathological Proteins

**Figure 3:**
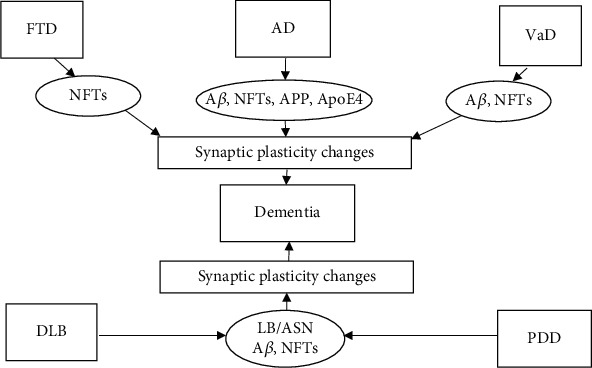
Changes in synaptic plasticity and the development of dementia in common dementia diseases. AD: Alzheimer's disease; FTD: frontotemporal dementia; VaD: vascular dementia; DLB: dementia with Lewy bodies; PDD: Parkinson's disease (PD) dementia; A*β*: *β*-amyloid; NFTs: neurofibrillary tangles; APP: amyloid precursor protein; LB: Lewy bodies; ASN: alpha-synuclein; ApoE4: apolipoprotein E.

Accumulation of soluble fibrillary oligomeric A*β* into extracellular A*β* plaques begins during the predementia phase in AD, long before synaptic loss and neurodegeneration can be detected. A*β* may be responsible for memory loss in AD patients by impeding synaptic plasticity and impairing activity of synaptic junctions (Figure 3, Table 1) [71, 72]. It has been shown that soluble A*β* oligomers may adversely affect synaptic structure and plasticity at very low concentrations [73]. The soluble oligomeric A*β* may also interact with several proteins, such as NMDA-type glutamatergic receptors and proteins responsible for the maintenance of glutamate homeostasis related to glutamate uptake and release. There are many indications that A*β* may directly affect NMDA receptor function. In neuroblastoma cell cultures (MES 23.5), administration of MK-801 or removal of extracellular Ca^2+^ resulted in a reduction of A*β*_1-40_-induced Ca^2+^ transients, NO production, and neurotoxicity [74]. Simultaneous incubation of hippocampal sections from experimental animals with A*β*_1–42_ oligomers strongly inhibited the induction of LTP in CA1 and dentate gyrus, but not NMDA receptor-independent LTP [75]. Importantly, NMDA and metabotropic glutamate 5 receptors (mGluR5) are in close proximity within postsynaptic complexes. Further, Renner et al. [76] showed that mGluR5 clustering as a result of interactions with A*β*_1–42_ oligomers causes a pathophysiologic increase in intracellular Ca^2+^ leading to synapse loss. An interaction of A*β* deposits with the PrPc prion protein has also been posited [77, 78]. A*β* oligomers have been shown to bind to PrPc in a way that allows PrPc to act as a receptor for A*β* or A*β*-derived diffusible ligand (ADDL) [79]. Seeing how PrPc mediates synaptic neurotoxicity [80], this phenomenon might be reversed via PrPc knockout [79]. All in all, the molecular mechanisms leading to synaptic damage in the presence of A*β* have not been comprehensively explained.

A lack of A*β* deposition and the presence of NFTs (which consist of intracellular proteins like tau and TDP-43) are neuropathological features of FTD [81]. Frontotemporal lobar degeneration- (FTLD-) tau is thought to account for 36-50% of all FTD degenerations. The most common FTLD-tau subtypes are Pick's disease, corticobasal degeneration, and progressive supranuclear palsy. Pick's disease accounts for 30% of FTLD cases. Pick bodies are the pathological hallmark of Pick's disease but may cooccur with Alzheimer's-type neurofibrillary lesions [64, 81].

Patients with DLB have faster cognitive decline than patients with AD dementia. While most patients with clinically diagnosed DLB have diffuse (neocortical) LB, some can have transient (limbic) LB [82]. Although both DLB and PDD are characterized by the deposition of cortical LB consisting of ASN, they can also exhibit A*β* plaques and NFTs [83]. While A*β* is deposited significantly more frequently in DLB than PDD, the degrees of NFTs or LB scores tend to be similar [84, 85]. In FTD and DLB, similar pathological proteins are found, but clinical pictures differ between the two diseases.

Genetic factors have also been implicated in aberrant changes to synaptic plasticity in AD. Among the genetic variants responsible for early-onset AD (EOAD) are amyloid precursor protein (*APP*), presenilin-1 (*PSEN1*), and presenilin-2 (*PSEN2*). While carriers of the deleterious apolipoprotein E, *APOE* E4 variant has an increased risk of developing AD and developing AD earlier in advanced age; ApoE4 is not considered a diagnostic biomarker for the disease [63, 64]. Nonetheless, both APP and ApoE4 are involved in the regulation of synaptic plasticity, synaptogenesis, synaptic expression, and neurodegeneration and likely factor into the said aberrant changes [86, 87].

The participation of *APOE* E4 in the development of vascular cognitive impairment has not been confirmed [88]. Moreover, the role of *APOE* in FTD is uncertain. Nonetheless, Rostgaard et al. [89] showed that *APOE* E4 may play a protective role in FTD caused by a mutation in the *CHMP2B* gene located on chromosome 3 (FTD-3). It has been shown that *APOE* E4 is more strongly associated with PD than DLB by localizing it in LB, oligodendrocytes, and the nuclei of melatonin-containing neurons [90].

### 5.2. Neurotrophins

**Figure 4:**
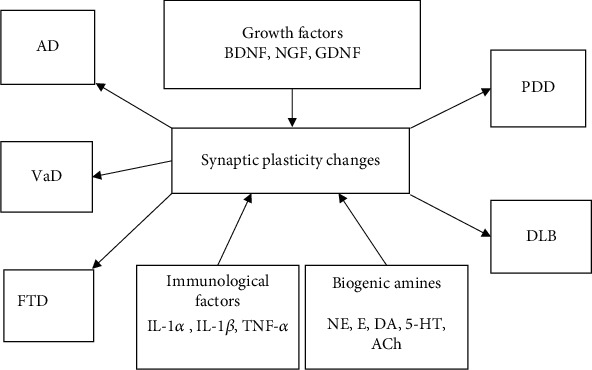
Changes in the level of neurotrophins and synaptic plasticity in the development of dementia diseases. AD: Alzheimer's disease; FTD: frontotemporal dementia; VaD: vascular dementia; DLB: dementia with Lewy bodies; PDD: Parkinson's disease (PD) dementia; BDNF: brain-derived neurotrophic factor; NGF: nerve growth factor; GDNF: glial-cell-derived neurotrophic factor; NE: norepinephrine; E: epinephrine; DA: dopamine; 5-HT: serotonin; ACh: acetylcholine; IL-1*α*: interleukin-1alpha; IL-1*β*: interleukin-1beta; IL-6: interleukin 6; TNF-*α*: tumor necrosis factor-alpha.

The capacity for synaptic plasticity in the adult brain is predicated upon a specific environment containing cell precursors, glial and endothelial cells, and substances with neuroprotective effects, including neurotrophic factors. Neurotrophins are divided into three classes: growth factors that stimulate cell proliferation and differentiation, cytokines that regulate the immune system, and neurotrophins that support neuronal differentiation and survival (Figure 4) [1].

#### 5.2.1. Growth Factors and Cognitive Function

Growth factors such as BDNF, NGF, and GDNF play an important role in the regulation of synaptic plasticity. BDNF regulates LTD and LTP, axon germination, dendritic proliferation, and neuronal differentiation [91]. Alterations in the level of BDNF may contribute to AD pathology [92]. Lee et al. [93] found reduced BDNF levels in both AD and MCI patients. Decreases in BDNF, NGF, and GDNF levels were also observed in 26 patients with moderate AD and in 62 with MCI [94]. Consequently, BDNF, NGF, and GDNF may represent markers of cognitive deterioration [92, 94].

The aforementioned literature results suggest that BDNF is among the factors responsible for synaptic integrity and hence cognitive functioning impacted in AD, but little is known about BDNF expression in other neurodegenerative diseases [95–101]. Ferrer et al. [102] evidenced unchanged BDNF expression in the frontal and temporal cortices of FTD patients. In contrast, Benussi et al. [103] demonstrated that three major neurodegenerative diseases leading to dementia, FTD, AD, and DLB featured reduced BDNF levels. It seems that in these neurodegenerative diseases, there is a similar molecular mechanism leading to an alteration of intracellular BDNF trafficking and signaling. This hypothesis has been bolstered by studies on the role of BDNF in AD, DLB, and FTD where the BDNF level may be regulated by ASN accumulation [104, 105]. Excessive ASN accumulation activates the protein Rab, which likely modifies BDNF endosomal trafficking. Rab belongs to a superfamily of small Ras GTPases which play an important role in the formation and segregation of endosomal membrane domains. Rab proteins have been suggested to play a key role in many stages of vesicle movement, including vesicle formation, cytoskeleton-mediated transport, and anchoring to target membranes. There are 8 subfamilies: Rab1, Rab2, Rab5, Rab6, Rab7, Rab8, Rab11, and Rab18, all of which localize to the cytosolic side of selected compartments in the internal membrane system [106]. GTP-activated Rabs interact with protein effectors, recruiting them to the appropriate membranes in a process which often initiates vesicular or organellar translocation [107].

#### 5.2.2. Immunological Factors and Neurodegenerative Diseases

IL-1*β* not only plays a role in inflammatory response but also mediates of a number of cell functions, including synaptic plasticity, thereby exerting an effect on hippocampus-dependent memory systems [108]. Together with BDNF, it may also protect neurons from damage caused by infection or injury [109]. IL-1*β* is a proinflammatory cytokine together with IL-1*α*, tumor necrosis factor-alpha (TNF-*α*), and interleukin 6 (IL-6). These are released in response to infection or damage and are centrally regulated through neurohumoral pathways [110]. It has been shown that A*β* induces the secretion of proinflammatory cytokines, such as 1L-1*β*, TNF-*α*, and IL-6, contributes to increased inflammation, and may lead to early microglial mediated synapse loss in the brains of AD patients [111–114]. The release of cytokines in the brain may lead to behavioral changes and cognitive and motor function deficits [109]. Studies in patients with MCI showed that lowering the level of proinflammatory cytokines may have a positive effect on episodic memory loss [115].

Inflammation is also implicated in the pathogenesis of FTD [116]. Nonetheless, Sjögren et al. [117] showed that elevated levels of inflammatory cytokines such as TNF-*α* and transforming growth factor-beta (TGF-*β*) do not necessarily occur in patients with FTD. ASN aggregate deposition in neurons and/or oligodendrocytes in PD and DLB is thought to alter microglial morphology and induce a proinflammatory microglial phenotype. In these disorders, as well as in VaD, cytokines such as TNF-*α* and IL-1*β*, which influence disease progression, are thought to be responsible for microglial activation [118]. Chronic inflammation may result in damage to the white matter, axons, and synapses and promote cognitive impairment [119].

### 5.3. Biogenic Amines

Chronic neuroinflammation may lead to neurodegeneration and subsequent decrease in NE levels [112]. In AD and FTD, decreased levels of NE and 5-HT have been linked to cognitive impairment [120, 121]. An excessive inflammatory response in brain may disrupt memory-related plastic processes by interfering with BDNF signaling [109]. However, there remains a paucity of knowledge on how the levels of biogenic amines change in AD and other dementive diseases [121]. PDD and DLB have been shown to be characterized by large changes in the central and peripheral levels of monoamines [122–124]. Furthermore, DLB and PDD patients have the same profile of neural dopaminergic and cholinergic deficit, whose severity rivals or even exceeds that seen in AD. Conversely, there is minimal evidence of cholinergic deficit in FTD. Still, cholinergic deficits are thought to be central in the development of dementia and cognitive and motor impairment [125]. An experimental VaD model showed a reduction in DA, 5-HT, and NE levels in the hippocampus, as well as a decrease in cortical 5-HT and 5-hydroxytryptamine 1A receptor (5-HT1AR) mRNA expression [126], suggesting that treatments aimed at increasing levels of single or multiple biogenic amines in neurodegenerative dementias may have therapeutic potential.

## 6. Pharmacotherapy of Dementia Diseases and Neural Plasticity

### 6.1. Acetylcholinesterase Inhibitors in Dementia Treatment

Although drugs for treating AD and other dementias have been developed, better and safer medicaments are continually being tested. Currently, acetylcholinesterase inhibitors (IAChE) are primarily used in the treatment of dementias. IAChE alleviate the cholinergic blockade associated with memory problems (Table 2), facilitate long-term hippocampal memory consolidation, and promote hippocampal induction of LTP [127]. IAChEs such as donepezil, rivastigmine, and galantamine represent first-line therapy for AD. Although several studies have questioned the effectiveness of IAChEs for AD, others have tied their use to a more plastic brain in the context of neurodegenerative changes. Spencer et al. [128] showed that donepezil is able to induce gamma oscillations in the CA3 region of the hippocampus in an experimental model and contribute to certain procognitive effects. In addition, Ginestet et al. [129] demonstrated that donepezil increased the number of ACh transporters and alleviated cholinergic neuronal degeneration in a rat experimental model.

The use of IAChE in other types of dementia with purportedly dysregulated cholinergic transmission has also been studied. Cholinergic deficit is more marked in DLB and PDD compared to AD. Not surprisingly, IAChEs allow for significant improvement in cognitive and behavioral functions in these dementias. However, only rivastigmine is approved for use in PDD due to ambiguous treatment inclusion criteria. Whether cholinergic deficit exists in VaD remains contested, and there is no evidence of cholinergic loss in FTD. Treatment responses in VaD are confounded by the wide heterogeneity of patients with cerebrovascular brain diseases. Nonetheless, donepezil has been shown to afford some cognitive benefits for patients with VaD, while galantamine might have therapeutic utility in mixed dementia, AD, and VaD. IAChE use may even be considered in FTD if cognitive and behavioral symptoms are severe [130].

### 6.2. Memantine in Dementive Diseases

Memantine (3,5-dimethyladamantan-1-amine) is another pharmacotherapeutic used to treat AD patients. Memantine regulates neuronal Ca^2+^ influx by acting as an uncompetitive NMDAR antagonist with moderate affinity for the channel [127, 131, 132].

In AD, NMDA receptors are continuously albeit partially activated, resulting in an impairment of synaptic plasticity. Total blockade of the NMDAR, however, would be equally if not more injurious. The medium affinity of memantine for NMDARs allows them to restore a physiological level of channel activation. Memantine is used in moderate to severe stage of AD and is thought to provide both neuroprotection and improve learning and memory deficits [105]. According to the meta-analysis performed by Matsunaga et al. [133], memantine monotherapy improved, albeit only slightly, cognitive function, behavioral abnormalities, and the capacity for performing daily activities and was well tolerated by patients with AD.

There is still no conclusive evidence for the effectiveness of memantine in FTD and other dementive diseases. A meta-analysis conducted by Kishi et al. [134] indicated that memantine improved symptoms of FTD slightly better than placebo and might be of therapeutic benefit for these patients. We have previously reported a case of early FTD wherein the individual benefited from memantine use [135]. Studies on the use of memantine in DLB and PDD patients demonstrated its significant impact on cognitive and motor function, attention, processing speed, executive functions, memory, and language [136]. Ritter and Pillai [137] showed that memantine provides little benefit in improving cognitive function but regulates glutaminergic transmission in VaD. Importantly, memantine does not cause extra pyramidal side effects [138].

### 6.3. Antiepileptic Drugs and Dementia

Epilepsy is more common in childhood but also often affects older adults (aged >65 years) [139–141]. Children and adolescents with epilepsy bear select cognitive dysfunctions that may hinder their learning. While about 30% of children with epilepsy develop partial cognitive deficits in the visual and auditory systems, such changes do not typically tread upon normal intellectual development. Still, in many cases, cognitive decline persists despite adequately managed seizures. In epileptic encephalopathies, frequent seizures exacerbate neurocognitive dysfunction by affecting synaptic plasticity or other elements integral to neural development, and prompt initiation of effective antiepileptic therapy is essential to reduce cognitive decline.

It has been shown that patients with AD are at increased risk of epilepsy [142]. Epileptic seizures in AD patients have been interpreted as secondary phenomena resulting from progressive neurodegeneration. The treatment of epilepsy consists of the administration of antiepileptic drugs (AEDs), which classified by mechanism of action include GABA-ergic drugs, Ca^2+^ channel inhibitors, sodium channel blockers, and glutaminergic drugs.

One of the causes of epilepsy is excitotoxicity caused by excessive glutamate. With glutamate considered as a common denominator, plastic processes could well be disrupted during epilepsy. Interestingly, Tekin et al. [143] showed that administration of the voltage-gated sodium channel blocker lamotrigine commonly prescribed for certain cases of epilepsy led to improvements in word recognition, naming, and depressed mood in patients with AD. This result accords with the observation that the APdE9 murine model A*β* pathology has abnormally functioning sodium channels. AEDs of the sodium channel blocker class include oxcarbazepine (OXC), levetiracetam (LEV), carbamazepine (CBZ), phenytoin (DPH), or valproic acid (VPA). Ziyatdinova et al. [144] showed that both CBZ and VPA reduced the number of frequent epileptiform discharges (EDs) but VPA was most effective in reducing EDs in an experimental murine model. VPA has been shown to exert a neuroprotective effect in AD [145] and induce neurogenesis both *in vitro* and *in vivo*. The use of CBZ in AD has been limited due to its low solubility, inefficient pharmacokinetic profiles, and numerous side effects. OXC was studied in 108 patients with AD and VaD but failed to yield improvement of agitation and aggression in these patients [146]. LEV treatment led to hippocampal remodeling, improvement of behavioral and synaptic dysfunction, and learning and memory deficits in hAPP mice [147].

Epilepsy also occurs more often among patients with other types of dementia such as FTD and DLB than in the general population. In these patients, there is an overlap of excessive network excitability with cognitive decline. In DLB, low doses of VPA may be effective in the treatment of agitation when combined with drugs such as quetiapine [148, 149], while the GABA-ergic drug, gabapentin (GBP), may offer relief for patients with FTD [150]. Zonisamide (ZNS), a Ca^2+^ channel inhibitor with broad spectrum activity, can be used in the treatment of epileptic seizures in PDD patients, while VPA should be avoided because it can worsen pyramidal symptoms [151]. In general, it seems that AEDs may prevent bouts of epilepsy and improve cognitive function in these dementias, all while displaying high tolerability and favorable pharmacokinetic profiles; however, further research into their full therapeutic effects is required.

### 6.4. L-dopa Therapy

In PD, dopaminergic neurons and DA are lost as a result of the degeneration of the substantia nigra. The strategy underlying PD pharmacotherapy is to compensate for DA deficiency and increased breakdown of the neurotransmitter in the brain or provide the body with drugs that stimulate DA receptors (D1 and/or D2). It has been shown that the substantia nigra is involved in synaptic plasticity and L-dopa administration may have an effect on plastic processes [152, 153]. Milosevic et al. [154] showed that reduced levels of substantia nigra plasticity may be associated with increased motor symptoms.

L-dopa is also used to help patients with DLB and PDD control motor symptoms, but the dose is often limited due to the fact that it may cause agitation, cognitive dysfunction or visual hallucinations [149, 155]. Therapeutic use of L-dopa has also been studied in other dementias accompanied by parkinsonian symptoms such as in patients with EOAD and FTD [156, 157]. However, Jang et al. [156] showed that parkinsonian signs in EOAD may be associated with factors other than presynaptic dopaminergic deficit, suggesting that L-dopa should not be indicated for these patients. Parkinsonian symptoms observed in patients with FTD are most often associated with the behavioral variant of FTD (bvFTD), for which L-dopa therapy does not generally afford therapeutic benefit. Further, aberrations in the cholinergic system as seen in both AD and VaD may stem in part from concomitant disturbances to the dopaminergic system. As L-dopa normalized the cholinergic system in AD but not VaD, its administration may help in distinguishing patients with AD or mixed dementia from patients with “pure” VaD [158].

### 6.5. Psychological Symptoms of Dementia Therapy

Behavioral disturbances and psychological symptoms occur in up to 90% of patients with dementia. They are recognized in patients with AD, FTD, PDD, DLB, and VaD. In the treatment of neuropsychiatric symptoms in these patients, several classes of drugs are indicated in addition to those already mentioned, namely, antipsychotic and antidepressant medicines [138, 150, 159].

Antipsychotics are often used to treat behavioral disorders including aggression and agitation and psychological symptoms of dementia, especially psychosis [138, 150]. They are often prescribed in combination with AD drugs and other psychotropic medicaments, e.g., antidepressants. However, their use in the treatment of psychological symptoms of dementia often leads to significant extra pyramidal side effects.

### 6.6. Other Drugs Used in Dementia Therapy

One AD disease-modifying treatment is the monoclonal antibody, aducanumab. It has been shown that aducanumab crosses the blood-brain barrier and selectively binds to A*β* aggregates. In patients with the prodromal phase of the disease and mild dementia, it reduced A*β* levels in proportion to the administered dose and duration of therapy. Moreover, AD patients receiving aducanumab showed significant benefits in cognitive functions including memory and verbal skills and orientation and reported an overall positive effect with the treatment [160]. Unfortunately, phase III trial outcomes for aducanumab were disappointing [161].

Pore-forming polymeric 1,3-alkylpyridinium salts (Poly-APS) may be effective in tauopathies such as AD and FTD [162]. Another drug used in cholinergic degeneration of cortical neurons and synaptic plasticity decline, ladostigil (TV-3326 (N-propargyl-3R-aminoindan-5-yl) methylmethylcarbamate), appears to be effective in AD and DLB. Its effectiveness also appears to be confirmed in synucleinopathies such as PDD [163]. Ladostigil has an inhibitory effect on both AChE and butyrylesterase (BuE) and thereby improves cholinergic transmission and monoamine oxidases A and B, whereby it likely affects dopaminergic transmission. The potential role of the steroid hormone, 17*β*-estradiol, on synaptic plasticity and hippocampal-dependent cognitive function was investigated in an experimental VaD model [164]. In this study, 17*β*-estradiol was administered at a low dose for the first 3 months after VaD was experimentally induced and demonstrated a long-lasting beneficial effect, which included significant neuroprotection of hippocampal CA1 neurons. Administration of this steroid also prevented damage to the myelin sheaths and oligodendrocytes of the hippocampus, as well as loss of dendritic spines in the CA1. Finally, treatment with low doses of this steroid reduced the levels of proteins responsible for the development of dementia, such as p-tau and A*β*_1-42_. The use of low doses of 17*β*-estradiol may be potentially promising therapeutics in chronic cerebral hypoperfusion and VaD.

## 7. Conclusions

The molecular mechanisms of dementia diseases including AD, FTD, DLB, PDD, and VaD especially at the locus of impaired synaptic plasticity are not yet fully understood. As such, further research into the complex mechanisms underlying neuronal degeneration and the complex interactions between vascular and neuronal cells in dementia is required. Leveraging new technologies from nanoscale to whole-brain imaging, proteomics and bioinformatics studies, neural network research and research into the roles of exosomes, cytokines, and microRNAs in the development of neuropathologies will undoubtedly aid our making progress in understanding the said queries. New studies might consider the discovery of new biomarkers, improving diagnostic strategies, and the development of novel therapeutics as concrete goals in the fight to improve the quality of life of the growing number of individuals affected by neurodegenerative diseases.

## Figures and Tables

**Table 1:** Molecular changes and clinical features in neurodegenerative diseases.

Biomarkers	Synaptic plasticity Molecular change/clinical features					References
	AD	FTD	DLB	VaD	PD/PDD	
Amyloid pathology A*β*	(1) A*β* deposition (2) Cognitive decline (3) Memory loss (4) Unprovoked seizures (5) Nonfibrillar oligomeric A*β* and dysfunction of synapse structure	(1) A*β* deposition is not a neuropathological feature	(1) A*β* much more than in PDD	(1) A*β* pathology less than AD (2) Cognitive decline	(1) A*β* pathology less than DLB	Jack et al. [64] Small [71] Minkeviciene et al. [72] Bang et al. [81] Chai et al. [83] Fujishiro et al. [84] Sabbagh et al. [85] Day et al. [46]
Tau pathology NFTs	(1) Tau aggregation (2) Neuronal damage (3) Apoptosis	(1) Tau degeneration (2) Abnormal protein deposition (3) Neuronal degeneration (4) Rapidly progressive dementia (5) Memory deficit	(1) Tau degeneration not associated with DLB (2) High Braak NFT (3) Dementia	(1) Tau pathology less than AD (2) Apoptosis	(1) Tau degeneration not associated with PD/PDD	Jack et al. [64] Kaufman et al. [65] Bang et al. [81] Graff-Radford et al. [82] Chai et al. [83] Fujishiro et al. [84] Day et al. [46]
Growth factors BDNF NGF GDNF	(1) Upregulate expression (2) Cognitive deficit	(1) BDNF unchanged expression (2) BDNF decreased level	(1) BDNF decreased level (2) BDNF level *α*-synuclein-accumulation dependent	(1) BDNF decreased level (2) Learning and memory deficit	(1) BDNF level *α*-synuclein-accumulation dependent	Budni et al. [92] Lee et al. [93] Ferrer et al. [95] Fang et al. [104] Spillantini and Goedert [105] Xing et al. [108]
Immune system IL-1*β* TNF-*α* IL-6 NO	(1) Inflammation (2) Cognitive deficit (3) Memory deficit	(1) Autoimmunity (2) Increased intrathecal production of cytokines	(1) Inflammation (2) Microglia activation (3) Progress of disease	(1) Inflammation (2) Glia activation (3) Cognitive impairment	(1) Inflammation (2) Microglia activation (3) Progress of disease	Patterson [109] Hong et al. [112, 113] Bossù et al. [115] Sjögren and Wallin [116] Sjögren et al. [117] Hoffmann et al. [118] Wang et al. [119]
Biogenic amines NE E DA 5-HT ACh	(1) Disturb level of biogenic amines (2) Cognitive impairment	(1) Disturb level of biogenic amines (2) Cognitive impairment (3) Serotonergic and noradrenergic deficiencies	(1) Disturb level of biogenic amines (2) Dopaminergic and cholinergic deficit (3) Dementia	(1) Decrease level of biogenic amine level (2) Cognitive impairment	(1) Disturb level of biogenic amines (2) Dopaminergic and cholinergic deficit (3) Dementia	Walsh et al. [114] Dringenberg [120] Vermeiren et al. [121] van der Zee et al. [122] Hong et al. [112] Dorszewska et al. [123] Klein et al. [125] Guo et al. [126]
Other factors *APOE* E4 LB Cathepsin D	(1) *APOE* E4 shorter disease duration, regulation of synaptic plasticity (2) Cathepsin D correlated with NFTs score	(1) *APOE* E4 uncertain/protective role in FTD-3	(1) *APOE* E4 shorter disease duration (2) LB toxic *α*-synuclein, cognitive symptom decline (3) Cathepsin D increased in two cortical regions, not correlated with LB	(1) *APOE* E4 not associated with vascular cognitive impairment	(1) *APOE* E4 LB pathology (2) LB toxic *α*-synuclein (3) Cathepsin D unchanged, not correlated with LB	Dorszewska et al. [63] Graff-Radford et al. [82] Chai et al. [83] Chai et al. [88] Rostgaard et al. [89] Rohn and Mack [90] van der Zee et al. [122]

AD: Alzheimer's disease; FTD: frontotemporal dementia; DLB: dementia with Lewy bodies; VaD: vascular dementia; PDD: Parkinson's disease dementia; A*β*: *β*-amyloid; NFTs: neurofibrillary tangles; BDNF: brain-derived neurotrophic factor; NGF: nerve growth factor; GDNF: glial-cell-derived neurotrophic factor; LB: Lewy bodies; IL-1*α*: interleukin 1-alpha; TNF-*α*: tumor necrosis factor-alpha; IL-6: interleukin 6; NO: nitric oxide; NE: norepinephrine; E: epinephrine; DA: dopamine; 5-HT: serotonin; ACh: acetylcholine; *APOE*: apolipoprotein E.

**Table 2:** Pharmacotherapy and clinical features of neurodegenerative diseases.

Drugs	Synaptic plasticity Clinical features improvement					References
	AD	FTD	DLB	VaD	PD/PDD	
IAChE Donepezil Rivastigmine Galantamine	(1) Small procognitive effects (2) Disease modification in animal studies	(1) Might have benefits in very severe cognitive and behavioral symptoms	(1) Improvement in cognitive and behavioral functions	(1) Consider when there is evidence of cholinergic deficit	(1) Improvement in cognitive and behavioral functions	Spencer et al. [128] Ginestet et al. [129] Noufi et al. [130]
Memantine	(1) Improves cognitive function, learning, and memory deficits (2) Small improvement	(1) Slightly improves clinical features	(1) Improves cognitive and motor function, memory, and language	(1) Little benefit in improving cognitive function	(1) Improves cognitive and motor function, memory, and language	Parsons et al. [131] Matsunaga et al. [133] Kishi et al. [134] Meng et al. [136] Ritter and Pillai [137]
AEDs LTG CBZ VPA OXC LEV ZNS GBP	(1) Positive effect on memory (2) Improved word recognition and naming (3) Reduced spontaneous seizures	(1) No clear data (2) Individuals have improved with gabapentin	(1) No clear data (2) Treatment of agitation	(1) No significant effect	(1) Treatment of epileptic seizures	Tekin et al. [143] Ziyatdinova et al. [144] Sommer et al. [146] Sanchez et al. [147] Hershey and Coleman-Jackson [149] Supasitthumrong et al. [150] Tombini et al. [151]
L-dopa	(1) No indication in parkinsonian syndrome of EOAD	(1) Consider L-dopa treatment in parkinsonism in FTD	(1) Limited use due to side effects worsens cognitive function	(1) No indication	(1) Improves synaptic plasticity	Hershey and Coleman-Jackson [149] Arias-Montaño et al. [152] Rajan et al. [153] Milosevic et al. [154] Picconi et al. [155] Jang et al. [156] Siuda et al. [157] Nardone et al. [158]
Antipsychotic/antidepressants	(1) Noncognitive disorders, in behavioral disorders and psychological symptoms of dementia	(1) Noncognitive disorders, in behavioral disorders and psychological symptoms of dementia	(1) Noncognitive disorders, in behavioral disorders and psychological symptoms of dementia	(1) Noncognitive disorders, in behavioral disorders and psychological symptoms of dementia	(1) Noncognitive disorders, in behavioral disorders and psychological symptoms of dementia	Legesse et al. [159] Ohno et al. [138]
Other drugs Aducanumab Poly-APS Ladostigil 17*β*-Estradiol	(1) Improves cognitive function, learning and memory deficits, and physiological and pathological symptoms	(1) Improves cognitive function and physiological and pathological symptoms	(1) Improves cognitive function	(1) Improves synaptic plasticity and hippocampal-dependent cognitive function	(1) Improves cognitive function	Sevigny et al. [160] Koss et al. [162] Youdim [163] Zhu et al. [164]

AD: Alzheimer's disease; FTD: frontotemporal dementia; DLB: dementia with Lewy bodies; VaD: vascular dementia; PDD: Parkinson's disease dementia; IAChE: acetylcholinesterase inhibitors; AEDs: antiepileptic drugs; LTG: lamotrigine; CBZ: carbamazepine; VPA: valproic acid; OXC: oxcarbazepine; LEV: levetiracetam; ZNS: zonisamide; GBP: gabapentin; EOAD: early-onset AD; Poly-APS: pore-former polymeric 1,3-alkylpyridinium salts.

## Abbreviations

A*β*: *β*-Amyloid
ACh: Acetylcholine
AD: Alzheimer's disease
AEDs: Antiepileptic drugs
ApoE: Apolipoprotein E
APP: Amyloid precursor protein
ASN: *α*-Synuclein
BDNF: Brain-derived neurotrophic factor
bvFTD: Behavioral variant of FTD
CBZ: Carbamazepine
CNS: Central nervous system
CT: Computer tomography
DA: Dopamine
DLB: Dementia with Lewy bodies
DPH: Phenytoin
EDs: Epileptiform discharges
EOAD: Early-onset Alzheimer's disease
E: Epinephrine
ERK: Extracellular signal-regulated kinase
FTD: Frontotemporal dementia
FTLD-tau: Frontotemporal lobar degeneration-tau
GABA: Gamma-aminobutyric acid
GBP: Gabapentin
GDNF: Glial-cell-derived neurotrophic factor
GLT1: Glutamate transporter
5-HT: Serotonin
5-HT1AR: 5-Hydroxytryptamine 1A receptor
IAChE: Acetylcholinesterase inhibitors
IL-1*α*: Interleukin-1alpha
IL-1*β*: Interleukin-1beta
IL-6: Interleukin 6
JNK: c-Jun N-terminal kinase
LB: Lewy bodies
LEV: Levetiracetam
LTD: Long-term depression
LTG: Lamotrigine
LTP: Long-term potentiation
MCI: Mild cognitive impairment
MRI: Magnetic resonance imaging
NE: Norepinephrine
NFTs: Neurofibrillary tangles
NGF: Nerve growth factor
NMDA: N-Methyl-D-aspartate
NO: Nitric oxide
OXC: Oxcarbazepine
PD: Parkinson's disease
PDD: Parkinson's disease dementia
PET: Positron emission tomography
Poly-ASP: Pore-former polymeric 1,3-alkylpyridinium salt
PPA: Primary progressive aphasia
PSEN1: Presenilin-1
PSEN2: Presenilin-2
RBD: EM phase of sleep
REM: Rapid eye movement sleep
ROS: Reactive oxygen species
SD: Semantic dementia
SOD: Superoxide dismutase
SPECT: Single-photon emission computed tomography
TGF-*β*: Transforming growth factor-beta
TNF-*α*: Tumor necrosis factor-alpha
VaD: Vascular dementia
VPA: Valproic acid
ZNS: Zonisamide.
